# Supplementary figures and images for: Association of Ligamentum Flavum Hypertrophy with Adolescent Idiopathic Scoliosis Progression—Comparative Microarray Gene Expression Analysis
Source: Int J Mol Sci. 2022 May 1;23(9):5038. doi: 10.3390/ijms23095038 (PMC9101523; doi:10.3390/ijms23095038)

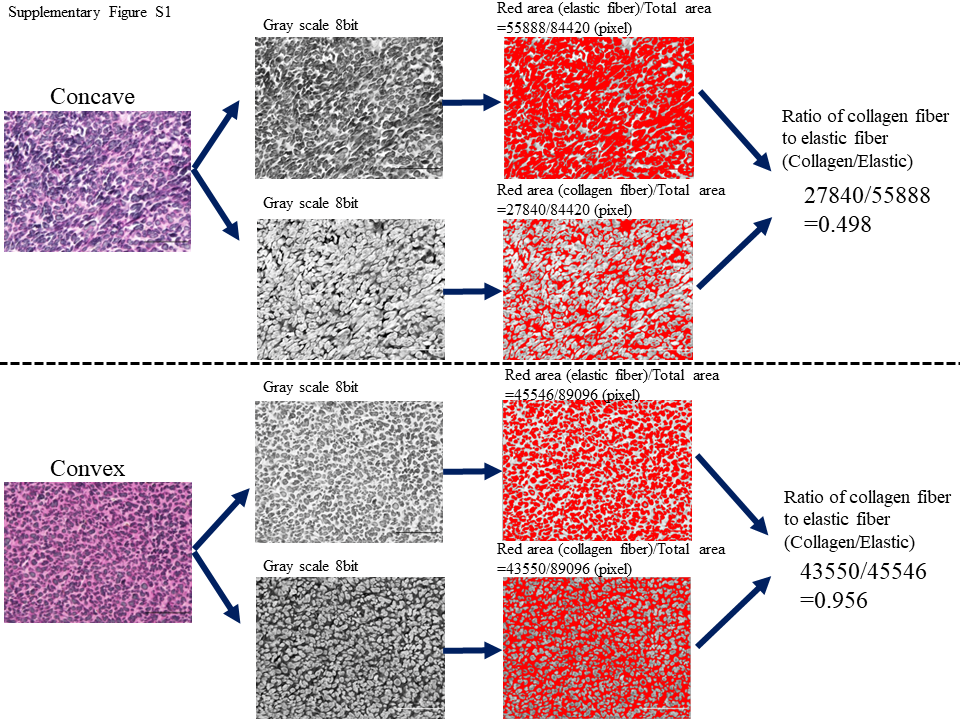

Supplement: Supplementary file 1 [file ijms-23-05038-s001.zip › ijms-1685007-supplementary.tif]
